# Translational Epidemiology: Developing and Applying Theoretical Frameworks to Improve the Control of HIV and Other Sexually Transmitted Infections

**DOI:** 10.1093/infdis/jiu559

**Published:** 2014-12-01

**Authors:** Helen Ward, Simon Gregson, Charlotte Watts, Geoffrey P. Garnett

**Affiliations:** 1 Department of Infectious Disease Epidemiology, School of Public Health, Imperial College London; 2 Department of Global Health and Development, Faculty of Public Health and Policy, London School of Hygiene and Tropical Medicine, United Kingdom; 3 Integrated Delivery Department, Bill and Melinda Gates Foundation, Seattle, Washington

The continued burden of disease caused by sexually transmitted infections (STIs), with 499 million cases of curable infections each year [1–3], constitutes a public health failure. Even in high-income countries, where extensive testing and treatment is available, STIs remain stubbornly endemic. It seems likely that this failure reflects our limited understanding of the complex individual, social, and cultural drivers of epidemics and of the interventions required in different contexts.

Mathematical models of STI and HIV transmission have been used extensively to understand transmission dynamics, and to estimate and predict the impact of interventions in diverse settings. At a population level, contact rate, transmission likelihood and duration of infectiousness determine the potential for spread of STI and the distribution of values for these three factors determines the extent of epidemics. Such theoretically derived insights can be tested through epidemiological studies exploring the risks of individuals acquiring infection and how population level measures of risk relate to observed prevalence of infection. However, such studies also show that a range of social, demographic and economic variables influence an individual’s risk of infection and that populations have different patterns of STI incidence and prevalence. Theoretical frameworks can be constructed to embody hypotheses about how these factors interact. Such frameworks allow us to structure our knowledge of STI epidemiology and understand the causal pathways linking variables.

Through a Wellcome Trust–funded research program titled “Developing and Applying Theoretical Frameworks in the Epidemiology of Sexually Transmitted Infections” (090285/Z/09/Z), we used theoretically driven statistical analysis, social science, and mathematical modeling to help improve our understanding of the transmission of STI. In this supplement, we draw together some of the outputs from this program and related collaborations.

One of the data sets generated or analyzed as part of the collaborations resulting from the program is used in the article by Davies et al [4]. Building on work showing a lack of good parameter estimates [5], the article provides additional quantification of the risk of pelvic inflammatory disease (PID) following chlamydial infection. In a population cohort of 74 000 women in Manitoba, Canada, the lifetime risk of PID was 55% higher following a positive test result, compared with a negative test result. Of importance for future intervention, there was heterogeneity in the PID risk, with girls aged <16 years with a repeat infection having an almost 5-fold greater risk than older women.

On the theme of heterogeneity, circular migration can play an important role in the dynamics of human immunodeficiency virus (HIV) acquisition and transmission, but not all migrants will be at risk of acquiring or transmitting HIV [6]. Using data from a household survey, Rai et al found that only 1 in 5 circular migrants reported behaviors characteristic of a sustaining bridge population. Interestingly, these men also had a heightened perception of their risk, a factor that could be exploited when designing prevention interventions.

The use of theoretical models allows explicit and focused interrogation of data [7]. A large cohort study in Manicaland, Zimbabwe, provided the basis for the next 2 articles [8, 9]. Eaton et al analyzed trends in different categories of partnership—concurrent, multiple, and polygynous—finding that, over a decade, the prevalence of each partnership declined as the HIV infection prevalence was also declining [8]. Elmes et al investigated factors affecting the prices charged by sex workers against the backdrop of Zimbabwe's collapsing economy. Client preferences dictated condom use, but unprotected sex attracted 40% higher payments than protected sex [9].

Gomez et al reviewed 36 studies on risk factors for gonorrhea acquisition [10]. A causal framework was developed, with different levels used to distinguish associations that are on the same causal pathway and those that are confounders. This framework can be used to guide hypothesis testing and, ultimately, in intervention design. For example, among sex workers, condom use was associated with infection, and condom use was better predicted by partner characteristics than by number of partners. This finding suggests that reducing the number of clients is less important than ensuring that condom use is high across all partnerships.

Another conceptual framework was developed to understand the phenomenon of seroadaptive behaviors in men who have sex with men (MSM), a major factor in the reemergence of other STIs, including lymphogranuloma venereum and syphilis [11]. Rönn et al found that high levels of awareness and testing are necessary, as are settings in which disclosure is facilitated, if seroadaptive behavior is to reduce harm.

The incidence of HIV infection among MSM remains high in many countries. White et al developed an individual-based model to quantify the number of HIV infections averted by behavior change in MSM with primary HIV infection for whom the infection was recently diagnosed [12]. Early treatment initiation can help reduce onward transmission, but because reducing the viral load takes some time to accomplish, good behavioral interventions will also be needed at this crucial time.

Finally, a perspective on changes over recent decades asks whether there is a convergence between behaviors traditionally associated with sex work and with noncommercial partnerships [13]. Population surveys show that large numbers of short-term sexual partnerships are not confined to commercial relationships and that sex work itself is more widespread and accepted in some places.

The articles in this supplement provide a useful demonstration of the ways in which theoretical frameworks can be further developed and used to help guide the development of combination interventions to control STIs.
